# Current Trend and Outcomes on Immunonutrition in Medical and Surgical Fields: An Updated Perspective

**DOI:** 10.21315/mjms2024.31.6.6

**Published:** 2024-12-31

**Authors:** Thai Hau Koo, Xue Bin Leong, Andee Dzulkarnaen Zakaria

**Affiliations:** 1 Department of Internal Medicine, Hospital Universiti Sains Malaysia, Kelantan, Malaysia; 2 School of Medical Sciences, Universiti Sains Malaysia, Kelantan, Malaysia; 3 Department of Surgery, Hospital Universiti Sains Malaysia, Kelantan, Malaysia

**Keywords:** immunonutrition, nutritional support, nutriproteomic, medical, surgical

## Abstract

Immunonutrition, which involves the targeted use of specific nutrients to enhance immune function and mitigate inflammation, has recently become a mainstay for both medical and surgical benefits. This review explores the evolution, mechanisms, and clinical applications of immunonutrition, with a focus on essential nutrients, such as omega-3 fatty acids, glutamine, arginine, and vitamins. These immunonutrients modulate immune responses, reduce pro-inflammatory cytokines, and support tissue repair. Clinical evidence indicates that immunonutrition reduces postoperative complications, shortens the duration of hospitalisation, and lowers the rate of infection, mainly in high-risk surgical patients and those with cancer or chronic diseases. In this regard, nutrients such as glutamine and omega-3 fatty acids have improved the nutritional status and recovery of cancer patients, while omega-3 fatty acids and antioxidant vitamins have exerted an anti-inflammatory effect, improving heart health in patients with cardiovascular disease. Immunonutrition has bright prospects in the management of infectious diseases, where certain nutrients, including vitamin D and zinc, aid in fighting immune defences and reducing the severity of infection. Future studies should investigate the molecular mechanisms underlying immunonutrition and its role in personalised nutrition. This could revolutionise dietary interventions based on genetic and proteomic profiling.

## Introduction

### Immunonutrition: Definition and Evolution

Immunonutrition may be defined as the use of specific nutritional substrates, known as immunonutrients, to modulate mechanisms involved in various immune and inflammatory pathways (1). These include omega-3 fatty acids, glutamine, sulphur-containing amino acids, antioxidants, arginine, and nucleotides, as a single entity or in combination (1). This was an incipient field of research with the aim of enhancing immune function through targeted nutritional interventions. It reinforces immune defences, reduces inflammation, and boosts health by exploiting specific nutrients. It is important to improve the prognosis of patients, especially in elective surgery, as it has been shown to reduce complications, decrease hospital length of stay, and reduce mortality (1). Immunonutrition refers to specific nutritional strategies that would support those with chronic diseases, patients undergoing surgery, and populations at risk of compromised immunity, such as elderly individuals and athletes, beyond the mere management of malnutrition (2). Clinical trials have demonstrated a decreased number of infectious complications and reduced hospital stay with immunonutrition, particularly in high-risk surgery patients, trauma victims, and critically ill patients. Recent studies have further explored its role in the tumour microenvironment, showing potential benefits for patients who have received chemotherapy and immunotherapy. A finding with possible implications for broader clinical practice with immunonutrition is that it improves patient response to certain anticancer therapies (3, 4).

The concept of immunonutrition has evolved over time from basic nutritional support to sophisticated targeted nutritional therapy. Nutritional support in the clinical setting begins with adequate caloric intake to prevent malnutrition. Early studies established a critical interrelation between nutrition and infection, in which nutrient deficiencies compromised host defences and accentuated disease outcomes. This relationship was formally codified by the 1968 World Health Organization (WHO) monograph, where this interrelation is unravelling if it is not complex enough, with further understanding of its interplay with food constituents. One critical avenue in this evolution is the identification and isolation of immunonutrients as a distinct class of compounds and their roles in modulating immune responses. Targeted nutritional therapies for specific clinical conditions have been developed and have made substantial progress (1).

Very early studies, dating back to the 1980s and the 1990s, examined the effect of specific nutrients on immune function and clinical outcomes, mostly in burn patients, for whom the metabolic demands were great and the potential benefits of immunonutrition were quite obvious (5). The major breakthroughs included administration of omega-3 fatty acids and glutamine during the perioperative period and their inclusion in systematic reviews and meta-analyses, further supporting the role of immunonutrition (1). Such developments have been a major milestone in the identification of specific nutrients, including glutamine and omega-3 fatty acids, which play a critical role in immune function.

Over the years, research has expanded to investigate the molecular mechanisms underlying these effects, including the interaction of nutrients with immune receptors, such as Toll-like receptor 4 (TLR-4) and nucleotide-binding oligomerisation domain 1 (NOD1). The development of specialised enteral feeds blending several immunonutrients with the formation of the International Society for Immunonutrition (ISIN) has propelled research and knowledge transfer in laying down the role of nutrition in immune health (6). These developments underline the requirement of shifting from general nutritional support to more fine-tuned evidence-based interventions to improve immune function and clinical outcomes.

The American Society for Parenteral and Enteral Nutrition (ASPEN) recommends immune-modulating enteral formulations for major elective surgeries, trauma, burns, and critically ill patients on mechanical ventilation; however, it does so with caution in cases of severe sepsis. Recent systematic reviews and professional society guidelines have established the role of immunonutrition in improving clinical outcomes, particularly in surgical and trauma patients (5). Although the results concerning cancer-related surgeries or other fields are very promising, the use of immunonutrition in orthopaedic and trauma surgery remains rather under researched and needs further investigation to arrive at appropriate conclusions (7).

This review seeks to update the existing knowledge in the domain of immunonutrition, with particular interest in bringing together new advances and recent findings in this area. This paper reviews the definition and importance of immunonutrition, its historical background, and the significance of this branch of nutrition in improving patient outcomes in the medical and surgical settings. This study underlined an important step in the evolution of the use of basic nutritional support that has moved toward targeted nutritional therapies, with broader implications in clinical practice for immunonutrition, and pinpointed milestones and breakthroughs in clinical practice.

## Immunonutrition in Medical Fields

### Mechanisms of Action

Immunonutrition is the potentiation of the immune response through specific nutrients exercised based on their anti-inflammatory action. Key immunonutrients include omega-3 fatty acids, vitamins A, C, D, and E, selenium, zinc, glutamine, and arginine, all of which greatly enhance immune function (8). They act by reducing pro-inflammatory cytokines, mainly tumour necrosis factor (TNF)-α and interleukin (IL)-6, promoting proliferation and immune activity, acting through T cells, B cells, and macrophages, and supporting gut barrier function through gut microbiota modulation.

For instance, omega-3 fatty acids resolve inflammation through specialised pro-resolving mediators, vitamins such as vitamin C support neutrophil function, and vitamin D enhances antimicrobial peptide production (9). Arginine is crucial for T-cell function and nitric oxide synthesis, which improves immune responses and promotes vasodilation. Glutamine is a fuel for rapidly dividing cells, including lymphocytes and enterocytes, and supports gut integrity and immune function. Immunonutrition also involves mechanisms of action in which dietary components influence immune responses by modulating cytokine production and functional capacities of various immune cells. Omega-3 polyunsaturated fatty acids (PUFAs) are known for their anti-inflammatory properties by incorporation into cell membranes, thus, influencing the production of inflammatory mediators, such as prostaglandins and leukotrienes, leading to decreased pro-inflammatory cytokines and potentially reversing immunosuppression in clinical settings. Polyphenols exhibit remarkable immunomodulatory effects through a mitochondria-centred multimodal approach, activating nutrient sensing via stress response pathways, regulating the mammalian target of rapamycin (mTOR)/adenosine monophosphate-activated protein kinase (AMPK) balance, and inhibiting the assembly of the NOD-, LRR- and pyrin domain-containing protein 3 (NLRP3) inflammasome (10). This multifaceted action results in the modulation of immune responses, promoting a less-inflamed, tolerogenic immunophenotype.

Sulphur-containing amino acids, such as cysteine and methionine, contribute significantly to the body’s antioxidant defences by maintaining glutathione levels, which protect cells from oxidative stress-induced damage and modulate the activity of transcription factors, such as nuclear factor kappa B (NF-kB), which controls the expression of numerous inflammatory cytokines. Collectively, these nutrients enhance the immune response, reduce inflammation, and improve clinical outcomes by decreasing infection rates and the length of hospital stay in various patient populations.

### Role of Immunonutrition in Disease Prevention and Management

Immunonutrition plays a crucial role in the prevention and management of chronic and infectious diseases by improving the immune function and reducing inflammation. For example, in type 1 diabetes mellitus (T1DM), polyphenols enhance mitochondrial function and inhibit immune cell infiltration into the pancreatic islets, thus, protecting against disease development (10). Polyphenols restore gut barrier function, induce autophagy, modulate immune cell metabolism, reduce symptoms, and maintain intestinal homeostasis in inflammatory bowel disease (IBD). In multiple sclerosis (MS), polyphenols show neuroprotective effects by modulating the immune response and decreasing neuroinflammation. Nutrient deficiencies can lead to compromised immunity and increased susceptibility to infections. For instance, vitamin D deficiency is associated with a higher risk of respiratory infections, whereas zinc deficiency can impair the development and function of the immune cells. Recent research has focused on the role of immunonutrition in the management of a wide range of diseases including cancer, cardiovascular diseases, and infectious diseases (6). By modulating the immune response and reducing inflammation, immunonutrients help manage conditions such as cancer, cardiovascular diseases, and infections.

Clinical trials have shown that preoperative immunonutrition reduces postoperative infections, shortens the length of hospital stay, and can also improve the survival chances of cancer and critically ill patients. For example, omega-3 fatty acids lower triglyceride levels and blood pressure, thereby, reducing the risk of cardiovascular disease. Clinical trials have shown that diets enriched with immunonutrients can reduce the incidence of infections and improve recovery rates (11). For instance, vitamin C supplementation showed some promise for pneumonia prevention and recovery from respiratory infections (9). Supplementation with vitamin D has been reported in clinical trials to decrease the risk and severity of respiratory infections, especially in those with low baseline vitamin D levels (9). Zinc deficiency results in impaired immune function and increased susceptibility to infection; therefore, zinc supplementation is of particular importance in older adults (9). A systematic review and meta-analysis of randomised trials for vitamin D supplementation confirmed its protective effects against acute respiratory infections, stressing that an adequate level of vitamin D is essential for immune health (9).

### Cancer

Immunonutrition, relative to cancer treatment, enhances the overall nutritional status and immune function of patients, which are usually compromised by the disease and its treatment. Nutrients such as glutamine and arginine have proven helpful in patients with malignancy because they enhance immune function and promote postoperative wound healing. Previous studies have established that immunonutrition reduces the occurrence of postoperative complications and hospital stays in patients with surgical cancer. For example, a meta-analysis demonstrated that immunonutrition significantly reduced infectious complications and wound infection rates in patients with malignancies (11). The potentially useful nutrients may include arginine, glutamine, omega-3 fatty acids, and fat-soluble vitamins A and E. Arginine and glutamine appear to play roles in the function and proliferation of T cells and macrophages related to antitumor immunity (8). Long-chain omega-3 fatty acids have been shown to reduce cachexia and enhance the quality of life of patients with cancer; this has been proven through randomised controlled clinical trials. A meta-analysis reported that vitamin E supplementation could improve immune function, thereby, reducing the risk of infection in cancer patients and improving overall outcomes.

Immunonutrition helps in the fight against cancer by supporting or enhancing immune cells to attack and kill cancerous cells, reducing treatment side effects and thus improving patient outcomes (10). Most current literature cites recent studies and meta-analyses arguing for the potential of polyphenols to improve anticancer therapies, reduce inflammation, and protect against treatment-induced oxidative stress. Glutamine, arginine, and omega-3 fatty acids are among the nutrients with great promise in patients with cancer. Glutamine helps maintain gut integrity and immune cell functions, which can be jeopardised during chemotherapy and radiation therapy (12). Arginine promotes T-cell activity by inducing nitric oxide production, which promotes tumoricidal activity. Recent clinical trials have suggested the potential of immunonutrients to reduce inflammation and aid recovery in patients with cancer after surgery, thereby, enhancing overall outcomes and quality of life (5). A systematic review of patients with head and neck cancer (HNC) found that radiotherapy and chemotherapy were undergoing continuous glutamine-enriched formula application, which led to a significant delay in the onset and reduced the severity of oral mucositis (13).

Moreover, arginine provides immune function and wound healing, which are critical components of cancer patients whose immune systems are often compromised and who seek enhanced recovery after surgery (ERAS) or radiotherapy. Omega-3 fatty acids may reduce inflammation and improve the nutritional status of cancer patients. Studies have shown that immunonutrition can improve or maintain nutritional and functional states and decrease the severity of treatment-related toxicities (13). For example, another important systematic review reported that one study reported significant improvements in weight, body mass index (BMI), and lean body mass in patients receiving immunonutrient-enriched formulas (14). These findings highlight the importance of incorporating immunonutrition into the comprehensive care of cancer patients to enhance treatment efficacy and improve quality of life.

### Cardiovascular Diseases

Immunonutrition plays a major role in cardiovascular health. Nutrients like omega-3 fatty acids, vitamins C and E, and selenium play a critical role in this regard. Omega-3 decreases triglyceride levels and inflammation, but improves heart health. Vitamin C also supports endothelial function, while vitamin E is an antioxidant that protects against oxidative stress. Selenium plays an important role in the biosynthesis of selenoproteins, which have antioxidant properties and can reduce inflammation (8). Clinical trials have recently demonstrated that these nutrients may have a positive effect on cardiovascular outcomes by modulating the immune response and reducing inflammation.

Omega-3 fatty acids are also important for cardiovascular health. They lower the levels of inflammatory markers, improve endothelial function, and lower the risk of atherosclerosis and cardiovascular events. Omega-3 fatty acids have been established through clinical trials as effective in the reduction of blood pressure and triglyceride levels in a way that adds to improved cardiovascular outcomes (11). Immunonutrition in the prevention and management of cardiovascular diseases is very important. Vitamins C and D have been associated with anti-inflammatory effects and improved endothelial function, and are key players in the prevention of cardiovascular diseases (9). Some evidence has already established that vitamin D deficiency increases cardiovascular risk factors, and vitamin D supplementation has been shown to enhance cardiovascular health. Modulation of zinc actions that reduce oxidative stress and inflammation further underlines better cardiovascular outcomes, and clinical studies have concentrated on the effects of zinc on heart health (9).

For instance, the Mediterranean diet, which is rich in polyunsaturated fatty acids, vitamins, and phytochemicals, improves inflammatory responses against atherosclerotic diseases (15). Omega-3 fatty acids, of which eicosapentaenoic acid (EPA) and docosahexaenoic acid (DHA) are important components, significantly reduce cardiovascular events by improving lipid profiles and decreasing inflammation. Vitamin D modulates immune responses and lowers vascular inflammation, underlining its role in cardiovascular health (16). Coenzyme Q10 improves endothelial function and lowers high-sensitivity C-reactive protein (hs-CRP) levels, providing indirect evidence of its anti-inflammatory potential. Therefore, these studies underscore the therapeutic potential of immunonutrients in the management of cardiovascular disease and highlight the need for further studies that can help optimise these interventions.

### Infectious Diseases

Immunonutrition enhances the immune defence against infections by providing nutrients that support the function of the immune system. For example, omega-3 fatty acids and vitamins C and D play important roles in reducing inflammation and enhancing immune cell functions. Vitamin D modulates the production of antimicrobial peptides, whereas vitamin C modulates neutrophil function and proliferation. Besides, selenium and zinc play critical roles in immune cell function and antibody production. Recent studies and systematic reviews have demonstrated the potential of immunonutrition in attenuating the severity and duration of, in this case, COVID-19 infection characterised by an overactive immune response (9).

Nutrients, such as glutamine and omega-3 fatty acids, improve gut barrier function and lower systemic inflammation, thus, playing a critical role in preventing and managing infections. Clinical studies have shown that immunonutrition may decrease the incidence of postoperative infections in critically ill patients by decreasing their incidence and improving the overall outcomes. A review indicated that immunonutrition decreased inflammatory markers and postoperative infectious complication levels; hence, immunonutrition is effective in patients undergoing surgery (9, 16). Vitamin C is well documented for its role in reducing the duration and severity of the common cold and respiratory infections. The immunomodulatory effects of vitamin D are particularly beneficial for the protection against respiratory pathogens. The involvement of zinc in immune cell function, with its deficiency linked to increased susceptibility to infection, underlines its importance in fighting infectious diseases (17).

Clinical trials and systematic reviews have provided consistent evidence that these nutrients play a role in reducing infection and improving recovery rates. Recent literature since the COVID-19 pandemic has shown that immunonutrition may have a potential role in improving patient outcomes. Vitamin C enhances the immune response prior to infection, helps in the neutralisation of pathogens during infection, and promotes tissue repair post-infection (17). Next, zinc inhibits viral replication, thereby, reducing the risk of inflammatory disease (18). Because of the high diversity and richness of the nutrient profile, the Mediterranean diet is recommended for systemic effects in the prevention and management of infectious diseases (19). Inclusion of these nutrients in the daily diet and supplementation during sickness can make a remarkable difference in enhancing the fighting ability of the immune system, reducing the severity of disease, and enhancing recovery (20).

## Immunonutrition in Surgical Fields

### Preoperative Nutritional Support

Preoperative nutritional support plays a very important role in the preparation of surgical patients for the stress of surgery and in the improvement of postoperative outcomes. Malnutrition is very common in gastrointestinal (GI) and HNC patients, and it increases the risk of postoperative complications by impairing the immune response and tissue healing mechanisms. Immunonutrition includes the administration of supplementary amounts of certain nutrients, such as arginine, omega-3 fatty acids, and nucleotides, in excess of standard protocols for nutritional support; it reportedly improves the immune response, reduces inflammatory reactions, and enhances wound healing, thus, improving surgical outcomes.

Research evidence proves that, in preoperative immunonutrition, postoperative complications are significantly reduced. For example, Yu et al. (21) conducted a prospective randomised controlled trial in 2024 on preoperative immunonutrition in patients with gastric cancer cachexia, establishing a decreased number of infectious complications and overall postoperative complications compared with standard enteral nutrition. These findings indicate that with immunonutrition, the postoperative levels of inflammatory markers were lower and immune markers were higher; this was found to correlate with a reduced length of stay in the hospital and less antibiotic use (21). Certain nutrients with immunomodulatory actions, such as omega-3 fatty acids, l-arginine, and nucleotides, can enhance the immune response, reduce inflammation, and promote tissue healing.

Recent clinical protocols, including the European Society for Clinical Nutrition and Metabolism (ESPEN) guidelines on clinical nutrition in surgery, suggest the routine use of immunonutrition in all high-risk patients (22, 23). ERAS protocols emphasise the role of perioperative nutrition, carbohydrate preload, and parenteral fluid restriction and suggest immunonutrition to help improve the overall nutrition and immune status of the patient (24). This approach has been supported by several guidelines from ASPEN and ESPEN and for the use of immunonutrition, especially in malnourished patients and major surgeries (24).

### Postoperative Recovery and Outcomes

The role of immunonutrition extends into the postoperative period, where it significantly affects recovery and outcomes, particularly, wound healing and infection reduction. Postoperative administration of immunonutrients helps maintain immune function, modulates inflammatory responses, and supports tissue repair. Studies have shown that postoperative immunonutrition, especially when continued from the preoperative period, can lead to lower rates of infectious complications and reduced hospital stays (25–27). Additionally, systematic reviews and meta-analyses, such as those by Guan et al. (25) and Takagi et al. (26), have demonstrated lower incidences of overall and infectious complications in patients receiving immunonutrition than in those receiving standard diet.

Current clinical protocols advocate the inclusion of immunonutrition in both preoperative and postoperative care to optimise patient outcomes. For example, enteral diets enriched with omega-3 fatty acids and arginine have been shown to improve oxygenation and reduce infection rates after thoracic esophagectomy (27). The inclusion of nutrients, such as arginine and omega-3 fatty acids, helps minimise scar formation and boost collagen synthesis, which is critical for effective wound healing (28). Postoperative administration of an enteral formula enriched with these nutrients reduces surgical site infections and other complications (29).

### ERAS Protocols

ERAS protocols are systematic, evidentially, and multimodally driven approaches to perioperative care that aim to optimise the recovery process of surgical patients. Since the initial adoption of colorectal surgery, ERAS protocols have been extended to a number of surgical subspecialties, such as thoracic and GI surgeries, including HNC surgeries, because of their effectiveness in improving patient outcomes (30–32). The basic principles of ERAS range from preoperative patient education to optimisation of health and nutritional status, minimally invasive surgical techniques, individualised anaesthesia, multimodal pain management, early initiation of oral nutrition, and early mobilisation after surgery (30, 32). As such, the protocols describe the standardisation of the administration of the perioperative care, limited use of opioids, proper fluid management, encouragement of ambulation in the early stages, and encouragement of early feeding. These interventions are adjusted according to the type of surgery and individual needs of the patients, with collaboration among surgeons, anaesthesiologists, nurses, dietitians, and others (30).

### Nutritional Supplements

Immunonutrition, a component of the ERAS, plays a crucial role in enhancing surgical recovery. It involves the integration of specific nutrients such as arginine, omega-3 fatty acids, glutamine, and nucleotides, which have immunomodulatory effects (33–35). These nutrients support the immune system, reduce inflammation, and promote wound healing (33, 35). For instance, preoperative nutritional assessment and correction of deficiencies, particularly in cancer patients, are critical because malnutrition is a significant predictor of poor outcomes. Integrating immunonutrition into ERAS protocols emphasises nutritional support to bolster immune response and promote healing. Current clinical protocols incorporate immunonutrition by reducing preoperative fasting, encouraging early postoperative feeding, and providing nutritional supplements tailored to patient needs. This approach decreases postoperative complications, shortens hospital stay, and improves overall patient outcomes by addressing nutritional deficiencies and supporting metabolic demands during the surgical recovery phase (30).

Nutritional supplements implemented in surgical patients as part of the ERAS protocols mainly include arginine and omega-3 fatty acids. Arginine is an amino acid that improves immune function and wound healing by promoting collagen synthesis (30). Omega-3 fatty acids also exert anti-inflammatory properties that appear to decrease the surgery-induced inflammatory response (30). It has been shown that dietary supplements may reduce the incidence of postoperative infections, promote recovery, and shorten the length of hospitalisation (33). For example, patients on an arginine-supplemented diet before surgery have been reported to have fewer postoperative complications and recover better than those who receive the standard diet (33). Similarly, data from a meta-analysis of perioperative immunonutrition in GI surgery showed a statistically significant reduction in postoperative complications, including infections, if arginine and omega-3 fatty acids were present in the nutritional regime (36). Although these data are very promising, evidence remains mixed, and large, well-designed independent trials are required to establish the definitive efficacy of immunonutritional supplements in surgical patients (37).

### Surgical Outcomes

The role of immunonutrition in reducing surgical morbidity and mortality is enormous, and many studies have indicated an enhancement in the quality of life of patients after surgery. The ERAS protocol combined with immunonutrition reduces postoperative complications such as infections and delayed wound healing (30). Immunotherapy has a positive impact on the enhancement of quality of life after surgery by hastening the recuperation process, thus, diminishing pain and discomfort and allowing the patient to return to normal activities much sooner (30). In 2016, Uno et al. (38) showed that immunonutrition significantly reduced the occurrence of postoperative infections and inflammatory marker levels in patients undergoing major hepatobiliary surgery. On the other hand, in some studies, such as Thornblade et al. (39), no significant differences in serious adverse events or length of stay were noted between patients who received immunonutrition versus standard nutrition. However, the odds ratios for postoperative infections and overall morbidity with immunonutrition were significantly decreased, underlining improvements in quality of life after surgery for the patients (34). While some evidence has shown benefits, the overall quality of the studies reviewed by Gupta and Senagore (40) had a number of deficiencies requiring better randomised controlled trials to draw conclusive evidence. However, considering that immunonutrition may improve postoperative recovery and the quality of life of patients, it must be applied rationally and according to individual requirements, as evidenced by current clinical practice (37, 41).

## Nutriproteomics in Immunonutrition

### Definition and Significance of Nutriproteomics

Nutriproteomics is a new area of science that uses proteomic tools to study nutrient-protein interactions at the biological system level. In other words, it links nutrition and proteomics to understand the effects of diet on protein expression and function in humans. It involves the application of proteomics in nutrition-related studies examining the effect of bioactive food ingredients on protein synthesis and their interactions with proteins. These interactions may be a post-translational modification or small molecule-protein interaction in nature and may manifest as changes in three-dimensional structure and function (42). Nutriproteomics considers data from the proteome of the host, proteomes of ingested food, and the intestinal microbial metaproteome, thus, taking the holism of nutrient-protein interactions into account. However, it should be noted that nutriproteomics may very well turn out to be one of the most powerful tools in the field of personalised nutrition, allowing for the identification of particular dietary proteins or peptides that can influence health outcomes and hold significant potential for targeted nutritional intervention strategies based on individual genetic and proteomic profiles. This dynamic interplay between nutrients and proteins is significant because it helps understand diet-related diseases, such as cancer, diabetes, and neurodegenerative diseases, potentially identifying biomarkers for diagnosing and treating these conditions (42).

### Technological Advances and Methodologies

Recent technological advances in the field of proteomics, which have enabled thorough proteomic analyses, have aided proteomic research in nutriproteomics. Such key methodologies include mass spectrometry, nuclear magnetic resonance, high-performance liquid chromatography, two-dimensional gel electrophoresis, protein microarrays, and immunological techniques, such as enzyme-linked immunosorbent assay. Techniques such as liquid chromatography-mass spectrometry and tandem mass spectrometry are classical for the identification of dietary proteins and peptides, as well as related post-translational modifications. One of the technologies coupled to mass spectrometry, nano-liquid chromatography, has increased sensitivity and resolution, allowing for the detection of low-abundance proteins (42). Additionally, bioinformatics tools are crucial for managing and interpreting the vast amounts of data generated by these technologies. Integrating these technologies facilitates the identification of dietary biomarkers, understanding of nutrient effects, and development of personalised nutrition strategies. Advancements in the field of bioinformatics and computational tools have advanced complex proteomic data analysis, providing a way to couple dietary components with specific health outcomes (41, 42).

### Biomarkers Identification

Biomarkers play a major role in both the diagnosis and follow-up of nutritional status and in the effects of dietary interventions. Changes in protein expression and modifications can be identified using proteomic technologies, which aid in the identification of biomarkers. Indeed, specific peptides from milk, such as caseins and whey proteins, have been described as biomarkers because of their role in immune modulation and antimicrobial activity (40, 42). Other studies have also identified specific proteins, including haemoglobin A1c (HbA1c), which is responsible for glucose control in diabetes, CA 72-4, which serves as an indicator for gastric cancer, and troponin, which aids in the diagnosis of myocardial infarction (43). Installing C-reactive protein (CRP) and ferritin have long been used as biomarkers for inflammation and iron levels, respectively. Mass spectrometry techniques have identified unique peptide signatures for certain allergens, such as those in milk, soy, and wheat, which can help in the diagnosis of food allergies (42). These biomarkers have potential applications in understanding diet-related effects on health and developing personalised nutritional therapies (42).

### Personalised Nutrition

Nutriproteomic profiles can be used to personalise nutrition based on unique genetic background, phenotype, and environmental factors that could potentially affect nutrient metabolism and health (4, 8, 17). Personalised nutrition concerns dietary plans tailored to optimise health by considering individual protein expression patterns (37, 42). This new field is expected to aid not only in preventing and managing chronic diseases but also in improving health, physical performance, and cognitive function (6, 27). There is an improvement in health outcomes, enhancement of disease prevention, and optimisation of performance through personalised nutrition. However, for this to be a widely available approach, certain challenges must be overcome: the integration of proteomic data with other omics data is complex, requires sophisticated bioinformatics tools and analysis, and personalised nutritional interventions remain costly (33–35). The potential for personalised nutrition to be fully exploited must engage ethical considerations that surround data privacy and access. Despite these challenges, personalised nutrition is promising for optimising health outcomes by aligning dietary interventions according to individual biological profiles, improving disease prevention and management, and enhancing our general understanding of nutrient efficacy (41–43).

## Conclusion

The targeted use of specific nutrients for modulating immune function and reducing inflammation has enormous potential benefits in a range of medical and surgical disciplines, defining immunonutrition itself. Among the key immunonutrients are omega-3 fatty acids, glutamine, arginine, vitamins, and polyphenols. These nutrients exert their synergy to improve immune responses, reduce pro-inflammatory cytokines, and support tissue repair. Clinical evidence indicates that immunonutrition reduces the occurrence of postoperative complications, shortens the length of hospital stay, and lowers infection rates, especially for those who have high-risk surgery, malignancy, or chronic diseases. In cancer care, specific nutrients, such as glutamine and omega-3 fatty acids, help to support immune function, improve nutritional status, aid recovery, and reduce treatment-related toxicity. Omega-3 fatty acids, along with vitamins C and E, play a role in attenuating inflammation and improving cardiac health in cardiovascular disease. There is also great promise for immunonutrition in the management of infectious diseases, whereby studies have been conducted to prove that nutrients such as vitamin D and zinc enhance immune defences and reduce the severity of infections.

Therefore, this immunonutrition should be integrated into standard care protocols by healthcare professionals in the management of patients undergoing major surgery, cancer treatment programmes, and those with chronic or infectious diseases. Preoperative and postoperative immunonutrition must be tailored towards improve immune status, reduce inflammation, and accelerate recovery. In surgical patients, there must be a preoperative nutritional assessment with inclusion of immunonutrients, such as arginine and omega-3 fatty acids, with the goal of improving outcomes, especially in malnourished or at-risk surgical patients. In this regard, continuous supplementation of immunonutrients in cancer care delays complications, particularly oral mucositis, and promotes recovery in these patients. Omega-3 fatty acids and antioxidant vitamins are recommended for heart health because they reduce inflammation and generally improve vascular function. During infectious disease outbreaks, adequate levels of vitamin D and zinc can bolster immune responses and mitigate disease severity.

Future research on immunonutrition should focus on elucidating the molecular mechanisms by which specific nutrients modulate the immune responses and inflammation. Emerging trends in nutriproteomics offer promising avenues for personalised nutrition strategies and tailoring dietary interventions to individual genetic and proteomic profiles. Ongoing studies are exploring the role of immunonutrition in enhancing the efficacy of cancer therapies, reducing cardiovascular events, and in managing chronic inflammatory conditions. Large-scale randomised controlled trials are needed to establish definitive guidelines for immunonutrition in diverse patient populations, including orthopaedic and trauma surgeries, which remains underexplored. The integration of advanced technologies such as proteomics and bioinformatics is crucial for developing targeted nutritional therapies that optimise health outcomes and support disease prevention and management.
